# Anatomical distribution of lower-extremity deep vein thrombosis and pulmonary embolism: a retrospective study with finite-element analysis of calf muscle-vein mechanics

**DOI:** 10.3389/fcvm.2026.1730081

**Published:** 2026-10-14

**Authors:** Xin Hong, Jia Hou, Xingyu Hong

**Affiliations:** Department of Vascular Surgery, China-Japan Union Hospital of Jilin University, Changchun, China

**Keywords:** deep vein thrombosis, pulmonary embolism, muscular calf vein, gastrocnemius, soleus, finite-element analysis

## Abstract

**Background:**

Muscular calf vein thrombosis is frequently detected by whole-leg ultrasonography, but its relationship with pulmonary embolism (PE) and the mechanical response of the intramuscular veins during calf-muscle contraction remain incompletely defined. We examined PE prevalence across anatomical categories of lower-extremity deep vein thrombosis (DVT) and used a structural finite-element model to quantify contraction-related venous compression and identify the regions in which deformation was greatest.

**Methods:**

This retrospective study included patients evaluated for a first episode of lower-extremity DVT between May and October 2022. DVT was classified as isolated muscular calf vein thrombosis, isolated axial calf DVT, isolated proximal DVT, or combined muscular calf and proximal DVT. PE was diagnosed by computed tomography pulmonary angiography or pulmonary perfusion imaging. Because several cells contained few observations, the four-group distribution was compared using a Fisher-Freeman-Halton exact test. A separate finite-element analysis used one normal computed tomography angiography geometry. Gastrocnemius contraction, soleus contraction, and co-contraction were modeled as structural loading conditions. Spatial stress and strain maps were examined to localize venous regions with the greatest structural response. No blood-flow, venous-valve, or thrombus model was included.

**Results:**

Among 160 patients with DVT, 30 had PE. PE was present in 14 of 125 patients with isolated muscular calf vein thrombosis [11.2%, exact 95% confidence interval (CI): 6.3%–18.1%], 1 of 4 with isolated axial calf DVT (25.0%, 95% CI: 0.6%–80.6%), 3 of 10 with isolated proximal DVT (30.0%, 95% CI: 6.7%–65.2%), and 12 of 21 with combined muscular calf and proximal DVT (57.1%, 95% CI: 34.0%–78.2%). The overall distribution differed across groups (exact *p* < 0.001). At the final recorded model-loading parameter, mean muscle von Mises stress was 21.51 kPa during gastrocnemius contraction, 1.16 kPa during soleus contraction, and 9.68 kPa during co-contraction. The corresponding changes in modeled venous-domain volume were −49.25, −17.07, and −64.08 mm^3^, respectively. The spatial maps showed nonuniform venous deformation, with the most prominent response concentrated around proximal branching regions and adjacent intramuscular venous segments.

**Conclusions:**

PE prevalence differed across the anatomical DVT categories and was highest when muscular calf and proximal veins were involved concurrently. The structural model provided direct evidence that calf-muscle contraction compresses deep veins and localized the regions of greatest deformation. These findings support targeted dynamic venous imaging, while effects on blood flow and thrombus behavior require prospective patient-specific validation.

## Introduction

Venous thromboembolism comprises deep vein thrombosis (DVT) and pulmonary embolism (PE), with PE representing its principal acute life-threatening manifestation (1, 2). The anatomical extent of lower-extremity DVT is clinically relevant because thrombus burden, risk of proximal extension, diagnostic sensitivity, and management differ between proximal and distal disease. Distal DVT includes thrombosis of the axial calf veins and the muscular calf veins, namely the gastrocnemius and soleal veins.

Muscular calf vein thrombosis is common in hospitalized populations, but reported associations with PE vary across cohorts and study designs (3–5). Clinical series have documented concurrent PE in patients with isolated calf DVT, whereas short-term outcomes of muscular and axial calf DVT have sometimes been similar under treatment. This heterogeneity argues against assuming that one calf-vein location invariably identifies the source of an embolus.

The soleal and gastrocnemius venous networks differ in branching pattern, drainage, and their relationship to the surrounding muscle (6, 7). Clinical and autopsy studies have described soleal thrombosis, proximal propagation along drainage veins, and PE in selected populations (6, 8–10). Although anatomical and structural models cannot by themselves establish thrombus formation or embolization, they can directly test whether muscle contraction transfers load to deep venous structures and can identify the venous regions most susceptible to deformation. We therefore combined a retrospective clinical analysis of PE prevalence across four DVT distributions with a finite-element analysis that quantified and localized venous compression during calf-muscle loading. The clinical and mechanical components were interpreted as distinct but complementary evidence streams.

## Methods

### Study design and setting

We performed a single-center retrospective study at the Department of Vascular Surgery, China-Japan Union Hospital of Jilin University. The study period was May 2022 through October 2022. The Ethics Committee of the China-Japan Union Hospital of Jilin University approved the study (2023111604). The participants provided their written informed consent to participate in this study.

### Eligibility and clinical variables

The source population comprised consecutive patients assessed for a first diagnosis of lower-extremity DVT during the study period. Eligible patients had complete whole-leg venous ultrasonography performed at the study hospital and a documented PE assessment by computed tomography pulmonary angiography or pulmonary perfusion imaging. Patients were not included if DVT or PE had been diagnosed previously, if the initial diagnostic assessment had been performed elsewhere without complete source imaging at the study hospital, if whole-leg ultrasonography was unavailable, if PE status could not be established from pulmonary imaging, or if the medical record lacked the variables required for anatomical classification.

Available variables were age, sex, DVT anatomical category, and PE status. Coagulation indices, thrombus dimensions, occlusion grade, treatment exposure, and longitudinal imaging were not available in a form suitable for patient-level analysis. The final number of included patients is reported in the Results.

### Sample-size and precision considerations

Because this study was a retrospective census of all eligible records within a prespecified six-month period, no recruitment-based *a priori* sample-size calculation was used. Precision was assessed with exact 95% confidence intervals for the observed PE proportions. The study was not designed or powered for pairwise subgroup comparisons or multivariable modeling; the widths of the subgroup confidence intervals were therefore used to guide the strength of interpretation.

### Anatomical classification of DVT

DVT was assigned to one of four mutually exclusive categories. Isolated muscular calf vein thrombosis comprised thrombus confined to the gastrocnemius and/or soleal veins. Isolated axial calf DVT comprised thrombus confined to the anterior tibial, posterior tibial, and/or peroneal veins. Isolated proximal DVT comprised thrombus involving the popliteal vein or a more proximal deep vein without documented muscular calf involvement. Combined muscular calf and proximal DVT comprised concurrent involvement of a muscular calf vein and a proximal deep vein. These categories correspond to the four groups recorded in the clinical dataset and were used without *post hoc* reclassification.

### CTA reconstruction and finite-element geometry

The finite-element component was based on one de-identified computed tomography angiography (CTA) dataset from an individual without DVT. The scan was acquired in the supine position and covered the abdomen and lower limbs; slice thickness was 1 mm. The in-plane resolution was not retained in the archived model record. Digital Imaging and Communications in Medicine data were segmented in Materialise Magics and exported as stereolithography files. Surface noise was removed and the geometry was smoothed in Geomagic before export in Initial Graphics Exchange Specification format.

The modeled region extended from the knee to the ankle and included the principal bony structures, gastrocnemius and soleus muscles and tendons, vascular structures, and surrounding soft tissue. The gastrocnemius and soleal veins were represented as solid, low-stiffness structures with a nominal diameter of 3 mm and paths derived from CTA anatomy. A muscle-fiber orientation of 16.2 degrees relative to the tibial axis was assigned. The model represented normal anatomy and did not contain thrombus. Use of a non-thrombosed geometry was intentional because the finite-element component was designed to isolate baseline load transfer from contracting calf muscle to adjacent deep veins. A thrombosed-vein model would require disease-specific lumen geometry, thrombus material properties, altered wall mechanics and contact, and intraluminal loading; it was therefore outside the scope of this baseline structural analysis.

### Material properties, mesh, and solution

The structural analysis was performed in COMSOL Multiphysics version 6.0 using a stationary solver. Linear elastic material behavior was assigned. Relaxed muscle was modeled with an elastic modulus of 8.2 kPa and a Poisson ratio of 0.49; contracting muscle was modeled with an elastic modulus of 200 kPa and a Poisson ratio of approximately 0.50. The increase in muscle stiffness with contraction is consistent with experimental elastography observations, although the numerical model parameters remained idealized (11). The archived parameter summary described the veins as low-stiffness solids but did not retain a separate numerical venous modulus.

The geometry was partitioned with a tetrahedral mesh containing 1,315,519 elements and 223,757 mesh vertices. The relative solver tolerance was 0.001, and each loading condition reached its final recorded solution after 15 iterations. A mesh-independence study was not performed.

### Loading conditions, boundary conditions, and model outputs

Three structural loading conditions were analyzed: gastrocnemius contraction with the soleus relaxed, soleus contraction with the gastrocnemius relaxed, and simultaneous contraction of both muscles. Contraction was implemented as a 20% external material strain in the active muscle domain. The archived result tables used a dimensionless continuation parameter, *para*, ranging from 0 to 0.10 in increments of 0.01. The inner aspect of the soleus was assigned a roller support to permit longitudinal sliding while limiting transverse displacement. Bone-related surfaces provided structural constraints. Muscle and adjacent venous structures were treated as ideally connected, with no relative sliding at the interface.

The analysis was purely structural. It did not include blood, intraluminal pressure, venous valves, fluid dynamics, fluid-structure interaction, or thrombus material. The principal quantitative outputs were mean von Mises stress in the active muscle domain, expressed in kPa, and change in the total modeled venous-domain volume, expressed in mm^3^. Negative volume change indicated compression relative to the unloaded configuration. Spatial maps of vascular stress and volumetric strain were assessed qualitatively to localize regions of greatest structural response within the modeled venous tree. Because the exported maps did not provide a consistent variable label and numerical maximum for every condition, they were used for anatomical localization rather than numerical comparison of peak values between conditions. Load increments were not treated as independent observations, and no inferential statistics were applied to finite-element outputs.

### Statistical analysis

Categorical clinical data are reported as counts and percentages. Exact 95% confidence intervals for PE proportions were calculated using the Clopper-Pearson method. The association between the four DVT categories and PE status was assessed with a two-sided Fisher-Freeman-Halton exact test because expected counts were small in the axial calf and proximal groups. No pairwise comparison was designated as primary, and no pairwise significance claim was made. Age and sex were summarized descriptively. Patient-level covariates were unavailable; consequently, multivariable adjustment was not performed. Statistical significance was defined as a two-sided *p* value below 0.05.

## Results

### Clinical cohort

A total of 160 patients met the eligibility criteria. There were 96 men and 64 women; age ranged from 20 to 90 years, with a mean of 64 years. PE was identified in 30 patients, corresponding to an overall prevalence of 18.8% (exact 95% CI: 13.0%–25.7%). For this fixed cohort, the exact 95% CI had a width of 12.7 percentage points. This supports descriptive estimation at the cohort level but not precise estimation in the small axial calf and isolated proximal subgroups.

Isolated muscular calf vein thrombosis was the largest anatomical category, accounting for 125 patients (78.1%). Four patients (2.5%) had isolated axial calf DVT, 10 (6.3%) had isolated proximal DVT, and 21 (13.1%) had combined muscular calf and proximal DVT. PE occurred in 14 of 125 patients with isolated muscular calf vein thrombosis, 1 of 4 with isolated axial calf DVT, 3 of 10 with isolated proximal DVT, and 12 of 21 with combined muscular calf and proximal DVT (Table 1). The PE distribution differed across the four categories (Fisher-Freeman-Halton exact *p* = 0.000020). The wide confidence intervals for the axial calf and proximal groups reflect their small sample sizes. The data did not support a statistically significant claim specifically comparing the combined group with the isolated proximal group.

**Table 1:** Pulmonary embolism according to anatomical distribution of lower-extremity DVT.

DVT category	Total, *n*	PE, *n*	No PE, *n*	PE, % (exact 95% CI)
Isolated muscular calf vein thrombosis	125	14	111	11.2 (6.3–18.1)
Isolated axial calf DVT	4	1	3	25.0 (0.6–80.6)
Isolated proximal DVT	10	3	7	30.0 (6.7–65.2)
Combined muscular calf and proximal DVT	21	12	9	57.1 (34.0–78.2)
Total	160	30	130	18.8 (13.0–25.7)

Data are *n* unless otherwise stated. The four-group Fisher-Freeman-Halton exact test yielded *p* = 0.000020. CI, confidence interval; DVT, deep vein thrombosis; PE, pulmonary embolism.

### Finite-element model performance

All three structural loading conditions reached the final recorded continuation value. The model and mesh are shown in Figure 1a, and the principal technical settings are summarized in Table 2. Because no mesh-independence or subject-specific validation study was available, the numerical results were interpreted as outputs from a single idealized geometry rather than population estimates.

**Figure 1:**
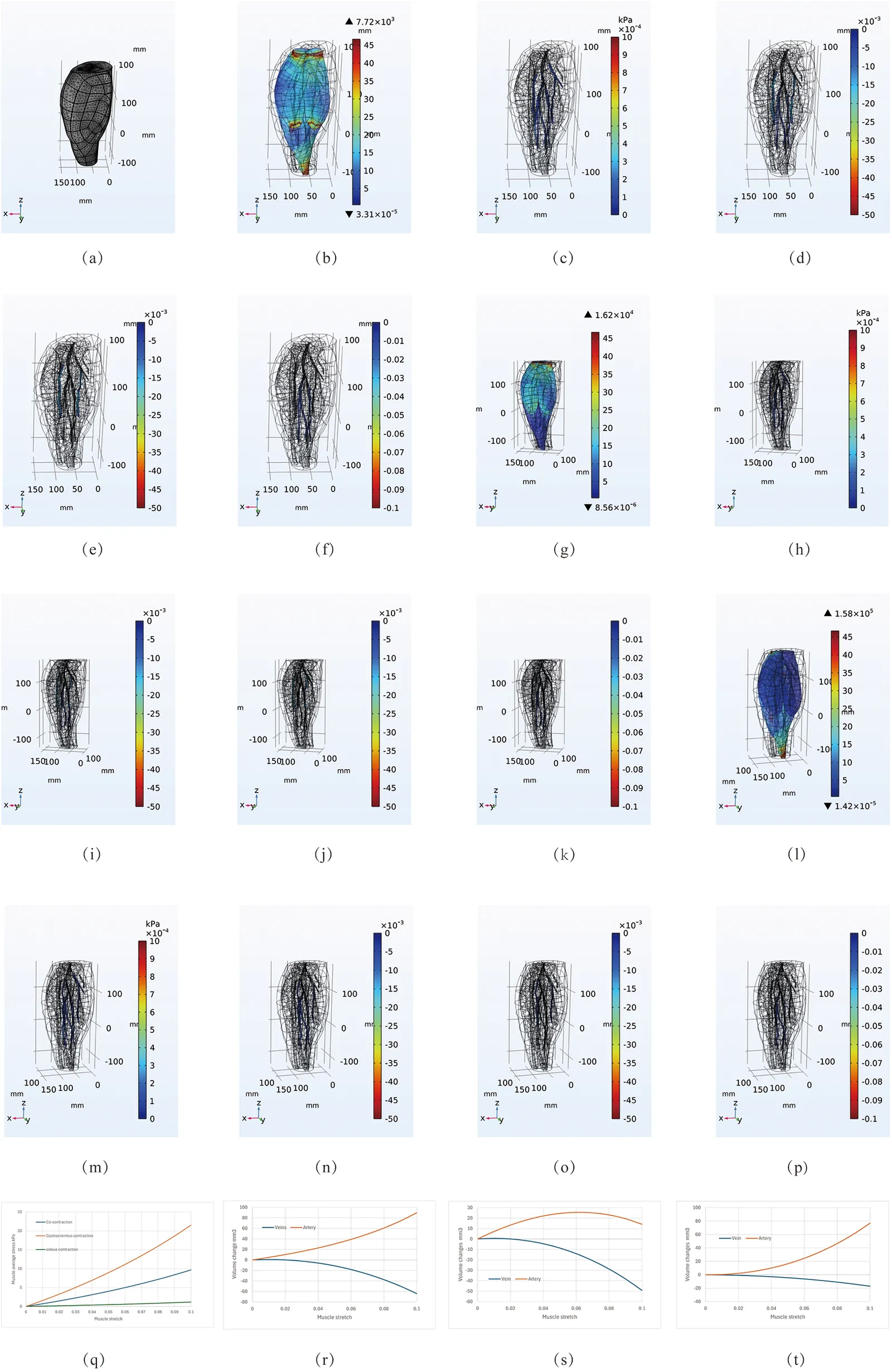
Finite-element model and structural response under three calf-muscle loading conditions. **(a)** Reconstructed geometry and tetrahedral mesh. **(b–f)** Muscle stress, vascular stress, and vascular strain distributions during gastrocnemius-soleus co-contraction. **(g–k)** Corresponding distributions during gastrocnemius contraction with the soleus relaxed. **(l–p)** Corresponding distributions during soleus contraction with the gastrocnemius relaxed. **(q)** Mean muscle von Mises stress across the recorded loading parameter. **(r–t)** Changes in venous and arterial domain volumes during co-contraction, gastrocnemius contraction, and soleus contraction, respectively. The spatial maps demonstrate a nonuniform vascular response and localize the most prominent stress and deformation to proximal branching regions and adjacent intramuscular venous segments. Quantitative interpretation in the text and Table 3 is limited to the muscle-stress and total venous-domain curves available in the archived source data.

**Table 2:** Principal settings of the finite-element model.

Model component	Specification
Geometry source	One CTA dataset from an individual without DVT; slice thickness 1 mm; supine acquisition
Geometry processing	Materialise Magics, DICOM to STL; Geomagic smoothing and noise removal, export to IGES
Solver	COMSOL Multiphysics 6.0.0.318; stationary structural analysis
Relaxed muscle	Linear elastic; Young modulus 8.2 kPa; Poisson ratio 0.49
Contracting muscle	Linear elastic; Young modulus 200 kPa; Poisson ratio approximately 0.50
Venous geometry	Solid low-stiffness structures; nominal diameter 3 mm; separate numerical modulus unavailable in archived summary
Muscle-fiber orientation	16.2 degrees relative to tibial axis
Contraction input	External material strain of 20% in the active muscle domain
Loading conditions	Gastrocnemius contraction; soleus contraction; co-contraction
Recorded continuation parameter	*para* = 0–0.10 in increments of 0.01
Interfaces and supports	Ideal muscle-vein connection without sliding; roller support on inner soleus surface; bone-related structural constraints
Mesh	Tetrahedral; 1,315,519 elements; 223,757 mesh vertices
Solver control	Relative tolerance 0.001; final solution reached after 15 iterations
Excluded physics	No blood flow, intraluminal pressure, venous valves, fluid-structure interaction, or thrombus
Model verification	Mesh-independence, parameter-sensitivity, and experimental validation were not performed

CTA, computed tomography angiography; DICOM, Digital Imaging and Communications in Medicine; DVT, deep vein thrombosis; IGES, Initial Graphics Exchange Specification; STL, stereolithography.

**Table 3:** Finite-element outputs at the final recorded loading parameter (*para*=0.10).

Loading condition	Mean muscle von Mises stress, kPa	Change in total modeled venous-domain volume, mm^3^
Gastrocnemius contraction	21.51	−49.25
Soleus contraction	1.16	−17.07
Gastrocnemius and soleus co-contraction	9.68	−64.08

Values are deterministic outputs from one normal-geometry model and are not population estimates. Negative volume change denotes compression relative to the unloaded configuration. The venous-domain result aggregates the modeled veins and does not distinguish the gastrocnemius and soleal venous networks.

### Magnitude and spatial localization of venous compression

Mean muscle von Mises stress increased monotonically across the recorded loading sequence in each condition (Figure 1q). At *para*=0.10, mean stress was 21.51 kPa during gastrocnemius contraction, 1.16 kPa during soleus contraction, and 9.68 kPa during co-contraction (Table 3).

The total modeled venous-domain volume decreased by the final loading point in all three conditions. The largest decrease occurred during co-contraction (–64.08 mm^3^), followed by gastrocnemius contraction (–49.25 mm^3^) and soleus contraction (–17.07 mm^3^) (Figures 1r–t and Table 3). These results demonstrate measurable transfer of muscle loading to the deep venous structures and show that the magnitude of compression depended on the activation pattern. The values describe the aggregate venous domain and do not separate the gastrocnemius and soleal venous networks.

Spatial maps showed heterogeneous muscle stress and localized vascular stress and compressive strain adjacent to the active muscle domains (Figures 1b–p). The most prominent high-response regions were concentrated around proximal venous branches and adjacent intramuscular segments rather than being distributed uniformly throughout the venous tree. Gastrocnemius contraction and co-contraction produced broader visible regions of vascular stress and deformation than soleus contraction alone. Thus, the model provided both a quantitative measure of overall venous compression and an anatomical map of the regions most susceptible to contraction-induced deformation. The maps do not provide measures of flow velocity, venous resistance, stasis, wall shear stress, or thrombus motion.

## Discussion

This study yielded two clinically relevant observations. First, PE prevalence varied across the four anatomical DVT categories in the retrospective cohort and was highest among patients with concurrent muscular calf and proximal DVT. Second, the single normal-geometry finite-element model directly demonstrated that calf-muscle loading was transmitted to deep venous structures, producing condition-dependent volume reduction and spatially localized regions of stress and deformation. The clinical and mechanical findings address different levels of evidence, but together they show why the muscular calf venous system warrants careful anatomical and functional assessment.

The clinical distribution was dominated by isolated muscular calf vein thrombosis, consistent with reports that muscular calf veins are frequently involved in hospital-acquired and isolated distal DVT (3–5). The PE proportion in the combined muscular calf and proximal category was 57.1%, but the confidence interval was wide and the group contained only 21 patients. This pattern is compatible with greater thrombus extent or proximal involvement being associated with concurrent PE, but the present data cannot determine whether an embolus originated in a muscular calf vein, an axial calf vein, or a proximal segment. Prior clinical studies have likewise reported variable PE frequencies in isolated calf and muscular-vein thrombosis (4, 8, 9). Differences in imaging protocols, inpatient case mix, thrombus size, and timing of pulmonary imaging may contribute to the variation.

The finite-element results add information beyond a single global measure of volume change. Gastrocnemius contraction generated the highest mean muscle stress at the final recorded load point, whereas co-contraction produced the greatest reduction in total venous-domain volume. Soleus contraction generated smaller changes in both measures. This condition-dependent response demonstrates that the location and combination of active muscle domains influence the magnitude of deep venous compression. In parallel, the stress and strain maps localized the strongest structural response to proximal branching regions and adjacent intramuscular venous segments. The combination of global volume reduction and regional mapping provides direct structural evidence of how contraction is transferred from calf muscle to the venous network. Anatomical studies describe the soleal venous system as a branching intramuscular network with defined proximal drainage routes (6, 7, 10), but the present model did not quantify separate soleal and gastrocnemius venous-domain volumes and therefore does not support a direct numerical comparison between the two venous systems.

The representative ultrasonographic images in Figure 2 provide an *in vivo* anatomical and functional context for the structural model. The soleal venous network appears as multiple communicating intramuscular channels and includes segmentally dilated venous spaces, whereas the illustrated gastrocnemius venous segment follows a comparatively simpler course; these appearances are consistent with established descriptions of muscular calf-vein anatomy (6, 7, 10). Dynamic color Doppler acquisitions also demonstrate detectable flow signals during gastrocnemius- and soleus-pump maneuvers. Together with the finite-element localization of high-response venous regions, these images support standardized dynamic ultrasonography as a practical approach for examining changes in muscular-vein caliber and flow signal during controlled calf-muscle activation. Because the images are illustrative and were not acquired under a quantitative velocity protocol, they do not establish comparative pump strength, valve competence, flow direction throughout the venous network, or thrombosis risk.

**Figure 2:**
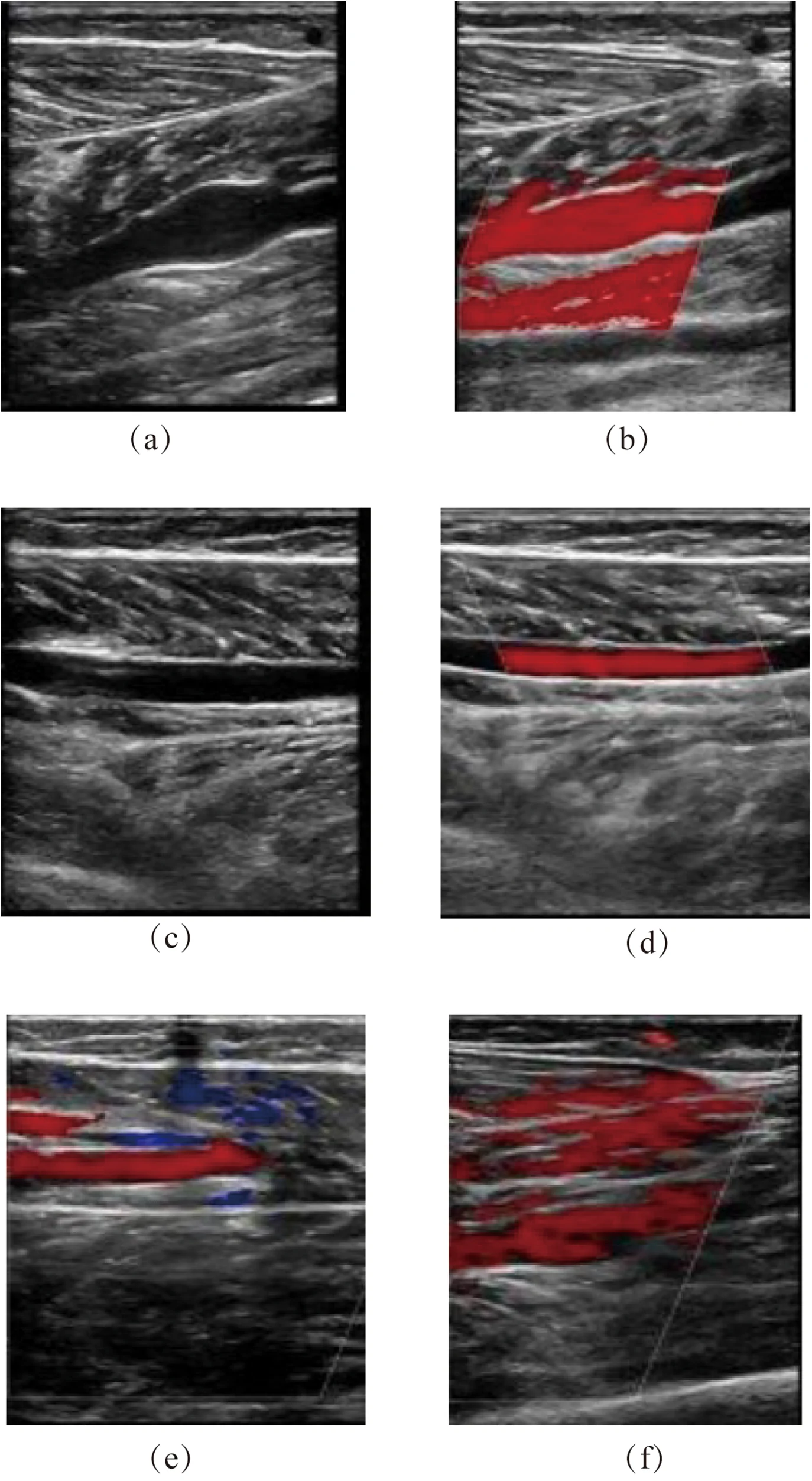
Representative ultrasonographic appearance of muscular calf veins and dynamic color Doppler response. **(a,b)** Gray-scale and color Doppler views of the soleal venous network, showing multiple communicating intramuscular channels and segmentally dilated venous spaces. **(c,d)** Gray-scale and color Doppler views of a gastrocnemius venous segment with a comparatively simpler course; the color signal indicates flow detected during image acquisition. **(e,f)** Dynamic color Doppler views obtained during gastrocnemius- and soleus-pump maneuvers, respectively. These images illustrate anatomical configuration and the feasibility of dynamic assessment; they do not quantify comparative pump strength, valve function, flow direction throughout the venous network, or thrombosis risk.

These structural findings should be interpreted within the defined scope of the model. The analysis did not simulate hemodynamics. A reduction in structural volume may alter venous flow *in vivo*, but the direction and magnitude of that effect depend on intraluminal pressure, valves, blood rheology, upstream and downstream boundary conditions, and the timing of muscle activation. None of these variables was included. Similarly, a model without thrombus cannot estimate thrombus formation, propagation, detachment, or embolization. Incorporating thrombus would materially change the model and would require disease-specific geometry, thrombus constitutive properties, vein-thrombus contact, and independent validation. The present model therefore serves as a normal-anatomy structural reference: it establishes the presence, magnitude, and anatomical distribution of contraction-induced venous compression, but it does not establish downstream effects on blood flow or thrombus behavior.

The clinical value of the finite-element evidence lies in identifying where and under which activation conditions venous compression is greatest. The regions highlighted by the model, particularly proximal branching areas and adjacent intramuscular venous segments, provide anatomically defined targets for dynamic ultrasonography and for prospective comparison of venous caliber at rest and during controlled calf-muscle activation. The results also support treating muscle activation state as an important variable when interpreting patient-specific venous deformation. Whole-leg ultrasonography should include careful assessment of the muscular calf veins when clinically indicated, because isolated distal and muscular-vein thromboses can be asymptomatic and can coexist with PE (12–14). Decisions regarding anticoagulation, serial ultrasonography, and duration of therapy should remain based on validated clinical risk assessment and current guidance (15–17). Future prospective studies should record coagulation markers, thrombus dimensions and location, treatment exposure, and serial pre- and post-treatment imaging. The present model provides a biomechanical basis for designing those functional studies rather than a stand-alone treatment rule.

Several limitations require emphasis. The clinical analysis was retrospective, single-center, and based on four markedly unequal groups. Only 30 PE events were observed, and two DVT categories contained very few patients. Patient-level data for coagulation markers, thrombus dimensions, occlusion, treatment, comorbidities, and follow-up were unavailable, precluding adjustment for confounding and analysis of thrombus evolution. The temporal relationship between DVT and PE imaging does not establish the source of embolization. The finite-element component used one normal CTA geometry and cannot represent anatomical variability or the altered mechanics of a thrombosed vein. The model used idealized linear materials, a nominal venous diameter, and simplified interface conditions; the archived record did not retain every tissue parameter. No mesh-independence, parameter-sensitivity, or experimental validation study was performed. Finally, the absence of blood flow, valves, intraluminal pressure, and thrombus prevents direct hemodynamic or embolic inference.

## Conclusion

PE prevalence differed across anatomical categories of lower-extremity DVT and was highest with concurrent muscular calf and proximal involvement. In the structural model, calf-muscle contraction transferred compressive loading to deep veins, caused measurable venous-domain volume loss, and localized the greatest deformation to proximal branching regions and adjacent intramuscular venous segments; the response depended on the activation pattern. These findings provide a biomechanical basis for targeted dynamic venous imaging, while effects on blood flow and thrombus behavior require prospective patient-specific validation.

## Data Availability

The raw data supporting the conclusions of this article will be made available by the authors, without undue reservation.
